# Certification of lay providers to deliver key population‐led HIV services in Thailand's National Healthcare System: lessons learned

**DOI:** 10.1002/jia2.25965

**Published:** 2022-07-25

**Authors:** Supabhorn Pengnonyang, Reshmie A. Ramautarsing, Surang Janyam, Tanachai Chaisalee, Pongthorn Chanlearn, Ravipa Vannakit, Praphan Phanuphak, Nittaya Phanuphak

**Affiliations:** ^1^ Institute of HIV Research and Innovation Bangkok Thailand; ^2^ Service Workers in Group Foundation Bangkok Thailand; ^3^ Rainbow Sky Association of Thailand Bangkok Thailand; ^4^ Mplus Foundation Chiang Mai Thailand; ^5^ Independent Researcher Bangkok Thailand

1

In Thailand, the HIV epidemic disproportionately affects men who have sex with men and transgender women, who account for more than half of new infections annually [1]. In 2015, the key population‐led health services (KPLHS) model was established to address low uptake of HIV services (testing, treatment and prevention) among these populations. KPLHS was designed by community members based on their needs and demands. Lay providers, themselves members of the communities they serve, are trained to deliver HIV and health services in key population‐led clinics established and operated by community‐based organizations (CBOs) [2]. These services include the provision of HIV counselling and testing, sexually transmitted infections (STI) testing and treatment, pre‐exposure prophylaxis (PrEP) and post‐exposure prophylaxis, and antiretroviral therapy (ART) for maintenance. KPLHS has been responsible for 55% of HIV testing in 2018, and 82% (12,748/15,546) of current PrEP users in 2021 in Thailand who are among key populations.

Initially, KPLHS was primarily funded through the US President's Emergency Plan for AIDS Relief and The Global Fund to Fight AIDS, Tuberculosis and Malaria. This was largely because CBOs were excluded from receiving reimbursement for clinical service delivery through the National Health Security Office (NHSO), which funds Thailand's Universal Health Coverage programme.

However, in 2016, NHSO began paying CBOs for HIV‐related reach and recruit (R&R) activities on a cost‐per‐head basis through an annual “social contracting” mechanism, totalling US $6 million [3]. Nevertheless, important barriers remained: (1) lack of perceived feasibility, value and contribution of lay providers to the national HIV response among local healthcare providers, national healthcare professional councils, and Ministry of Public Health (MOPH) and NHSO leaderships, (2) limitation of reimbursement to R&R‐related services and (3) the need to reapply for the contract every year.

Facing declining international investments, and recognizing that key population‐led organizations were ideally positioned and equipped to lead the HIV response, the Institute of HIV Research and Innovation (IHRI) and local CBO leaderships employed a long‐term, multi‐stakeholder, multi‐component strategy to reaffirm CBOs’ organizational capacity and enable certification of lay providers at the national level. The strategy aimed at providing direct reimbursement to CBOs, integration in the national healthcare system, and ultimately sustainability of KPLHS [4, 5]. The effort was supported by the United States Agency for International Development through ENGAGE and LINKAGES Thailand, together with UNAIDS. The strategy comprised the following components (Figure 1):

1. Development of training modules and administrative systems by IHRI, to facilitate certification of lay providers in HIV counselling, sample collection and conducting point‐of‐care tests for HIV and STI, and dispensing HIV‐related medications as prescribed by doctors (e.g. PrEP).
2. Development of a manual for quality standards for HIV service delivery, including key compentencies for lay providers, by IHRI in collaboration with The Department of Disease Control, MOPH and the Thailand MOPH—U.S. CDC Collaboration, to ensure the quality of services provided can be formally assessed and assured.
3. Identification of champions from among CBO leadership, to facilitate partnership with IHRI and reciprocal learning.
4. Establishment of a coalition for collaboration and joint advocacy efforts, including IHRI, CBO leadership, UNAIDS and LINKAGES Thailand.
5. Organization of a series of high‐level policy and advocacy dialogues with MOPH, central and regional NHSO offices, hospitals and provincial health offices, to raise awareness and generate support for task shifting to lay providers.
6. Presentation of scientific findings from KPLHS and its impact on the national HIV response, and conduct and facilitate site visits for policy makers to demonstrate the effectiveness and feasibility of the model, to assist with policy decisions.

**Figure 1:**
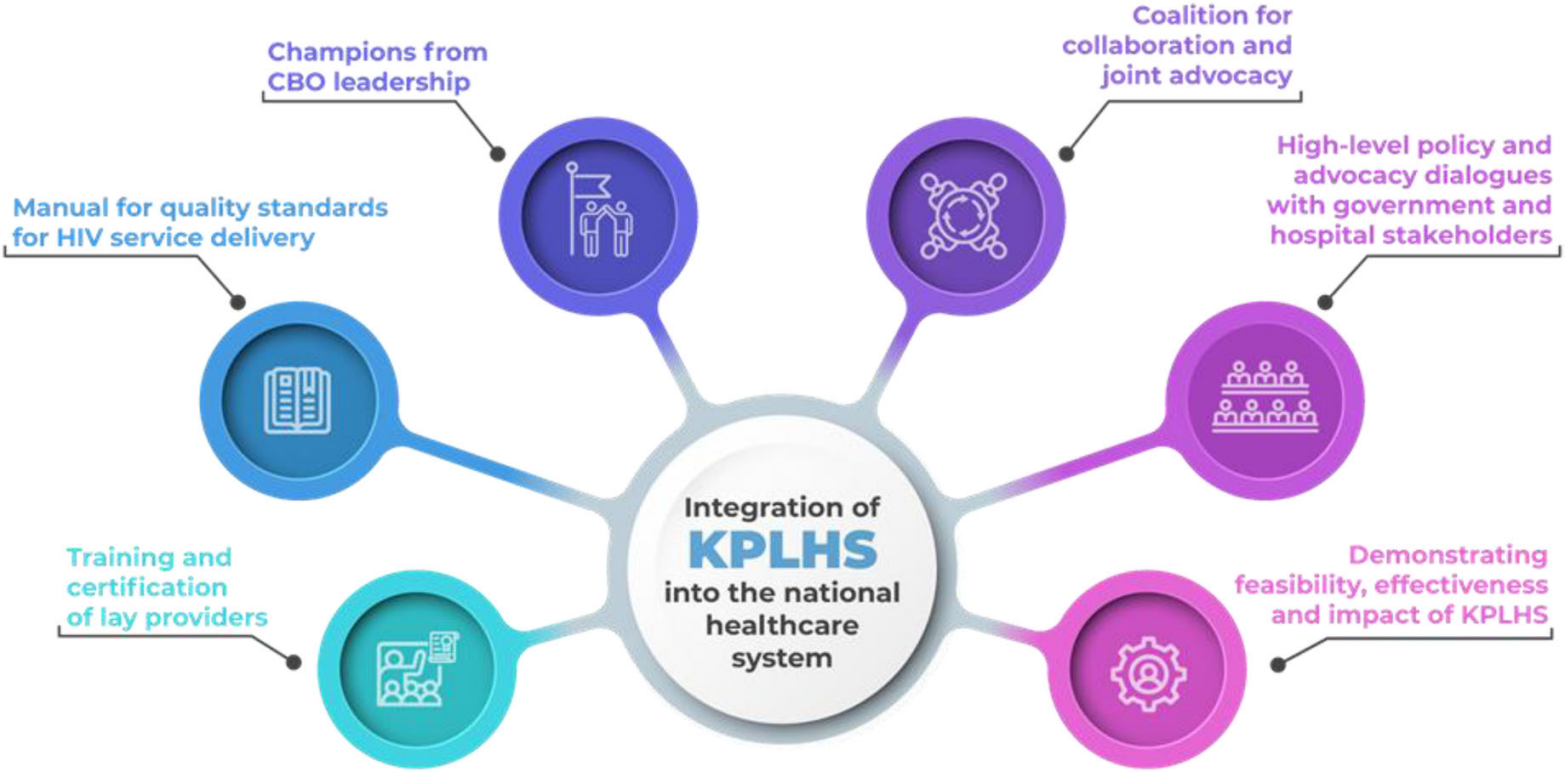
Components of a long‐term, multi‐stakeholder strategy to integrate key population‐led health services in the national healthcare system in Thailand. Abbreviations: CBO, community‐based organization; KPLHS, key population‐led health services.

In 2018, NHSO initiated an indirect reimbursement scheme for HIV testing and PrEP services conducted by certified lay providers, through which CBOs were paid via public hospitals. This scheme allowed more financial contributions to KPLHS, but faced key challenges: lack of acceptance of, and confidence among local public hospital leadership in the quality of services delivered by lay providers, resulting in difficulty partnering with them, and complications in reaching financial agreements.

As a result of our ongoing advocacy and demonstration of successful implementation, MOPH issued a decree in 2019, allowing certified lay providers to deliver high‐quality services (HIV counselling, sample collection for HIV and STI testing, conducting rapid/point‐of‐care tests and dispensing ART and PrEP as prescribed by doctors), signifying an important advance in regulatory reform to legalize lay providers to perform these services [6]. Furthermore, NHSO increased investments in local CBOs implementing KPLHS through social contracting for R&R and indirect reimbursement for HIV testing, from US$ 167,000 in 2016 to US$ 914,000 in 2019 [7].

The coalition among IHRI, CBOs and UNAIDS continued to engage in high‐level policy discussions engaging national healthcare professional councils, to obtain the endorsement of the KPLHS standards, training modules and certification system by the MOPH. This was the key step to advance direct reimbursement from NHSO to CBOs in order to address the bottleneck previously experienced with indirect reimbursement through public hospitals, and to expand it to other services, such as PrEP. In September 2021, the certification of lay providers and CBOs by MOPH was implemented at the national level. From September 2021 to May 2022, the number of certified lay providers increased from 199 to 263. Eighteen CBOs were certified in 2021 and 32 CBOs have already applied for certification in 2022. MOPH‐certified lay providers and CBOs, as described above, completed NHSO's requirement for direct reimbursement and activated its implementation.

Although Thailand's MOPH now recognizes IHRI's certification system, lay providers who have previously been certified by IHRI still need to take an additional MOPH examination to achieve national certification. HIV testing and PrEP have not yet been included as part of the national certification and remain reimbursable only through public hospitals. The programme partners continue to be committed to implementing a strategy in order to achieve harmonization of examination requirements and inclusion of HIV testing and PrEP in the certification system to facilitate direct reimbursement of these services.

MOPH and health professionals have now recognized that trained lay providers can strengthen existing HIV services in Thailand. The strategy has been successful in reaffirming feasibility and generating political and legal support, resulting in certification and integration of KPLHS into the national healthcare system. We have learned the following key lessons:

1. We need to move from community engagement to community leadership in designing KPLHS, demonstrating its feasibility, and facilitating certification and integration in the national healthcare system of KPLHS. They have dedicated themselves to drive KPLHS implementation since its inception, and overcame substantial initial resistance from health authorities. These community leaders are key contributors to policy changes, with powerful voices across negotiating platforms. Through their expertise and contribution, these champions have cemented their roles in policy and advocacy discussions at the national level in Thailand.
2. By demonstrating the increased uptake of HIV testing and PrEP among key populations and conducting ongoing advocacy activities, we have successfully turned yesterday's “not feasible” into today's successful KPLHS programmes. We need to rethink who gets to decide whether a programme is feasible and how this is ultimately defined.
3. Initial international funding can be leveraged to generate data on programme effectiveness, feasibility and impact in order to facilitate high‐level policy and advocacy discussions to ensure transitioning to domestic financing and sustainability.
4. Building coalitions with multiple domestic and international stakeholders is essential to defend, negotiate and advocate with transparency, sincerity and persistence, in order to overcome various regulatory and policy barriers. Community leadership in co‐delivering services has confronted health professionals who traditionally were placed at the top of a hierarchy. The client‐centred design also challenged paternalism where health professionals commonly restrict clients’ capacity to make own informed choice. A beneficial policy climate among coalitions is, therefore, crucial in the process especially in Thailand, where hierarchy and paternalism characterize its healthcare system and can delay the advancement of innovative HIV service delivery models.

## COMPETING INTERESTS

All authors declare no competing interests related to this work.

## AUTHORS’ CONTRIBUTIONS

SP, SJ, TC, PC, PP and NP designed, led and implemented strategies. SP and RAR wrote the first draft. SJ, TC, PC, PP and NP reviewed, revised and provided feedback on the draft. RAR and NP revised the following drafts. NP revised the final draft, which was reviewed and approved by all authors.

## FUNDING

This work was supported by the USAID and US President's Emergency Plan for AIDS Relief (PEPFAR) through the Linkages Across the Continuum of HIV Services for Key Populations cooperative agreement (LINKAGES) and the Meeting Targets and Maintaining Epidemic Control (EpiC) managed by FHI 360 and the USAID Community Partnership (ENGAGE) managed by IHRI.
